# Respiratory support with nasal high-flow therapy helps to prevent recurrence of postoperative atelectasis: a case report

**DOI:** 10.1186/2052-0492-2-3

**Published:** 2014-01-14

**Authors:** Yasuyuki Suzuki, Yasushi Takasaki

**Affiliations:** Department of Anesthesia, Uwajima City Hospital, 1-1 Goten-machi, Uwajima, Ehime, 798-8510 Japan

**Keywords:** Nasal high flow, Postoperative respiratory complication, Atelectasis

## Abstract

Postoperative atelectasis should be avoided in surgical patients with impaired pulmonary function. Nasal high-flow (NHF) therapy delivered by the Optiflow™ system (Fisher & Paykel Healthcare Ltd., Auckland, New Zealand) is a new, simple device that supplies heated and humidified oxygen gas at >30 L/min via a large-bore nasal cannula. We herein describe a case in which respiratory support with NHF therapy was useful for the prevention of postoperative atelectasis recurrence. A 67-year-old man with an upper digestive tract perforation underwent emergency laparoscopic surgery. He appeared malnourished because of severe untreated diabetes mellitus. The proposed surgery was uneventfully completed. On postoperative day (POD) 5, he experienced massive atelectasis of the left lower lobe with desaturation to <90%. After restoration of normal oxygenation by tracheal suction and postural drainage, noninvasive positive-pressure ventilation (NPPV) at a continuous positive airway pressure (CPAP) of 8 cm H_2_O was conducted to prevent repeated atelectasis. Fifteen hours after the cessation of NPPV on POD 7, he developed recurrence of massive atelectasis. Bronchoscopic suction removed a mucous plaque in the tracheobronchial tree, and NHF therapy at 40 L/min was subsequently performed, delivering a low level of CPAP instead of NPPV. Under the respiratory support with NHF therapy, his condition was more stable than with NPPV, and his respiratory rehabilitation continued uneventfully. In addition, the NHF therapy delivered optimally humidified gas, which improved the bronchial secretion quality. No further atelectasis occurred throughout the remaining stay in the intensive care unit. We conclude that respiratory support with NHF therapy may contribute to the prevention of postoperative atelectasis by delivering CPAP in combination with progressive respiratory rehabilitation.

## Background

Atelectasis is one of the most frequent respiratory complications after surgery. If massive atelectasis occurs postoperatively, the patient may require prolonged mechanical ventilation, worsening the outcome. Prevention of postoperative atelectasis is an important strategy, especially when managing patients with impaired preoperative pulmonary function. Nasal high-flow (NHF) therapy delivered by the Optiflow™ system (Fisher & Paykel Healthcare Ltd., Auckland, New Zealand) is a new, simple device that supplies heated and humidified oxygen at a high flow rate via a large-bore nasal cannula. The supplied gases contain a saturated water content of 44 mg H_2_O/L at 37°C; the humidity is optimal for maintenance of mucociliary function in the peripheral airways. NHF therapy reportedly delivers low-level positive airway pressure in contrast to conventional oxygen face masks. Therefore, it may be an effective supplementary respiratory support technique for the prevention of postoperative atelectasis. We herein describe a case in which respiratory support with NHF therapy helped to prevent the recurrence of postoperative atelectasis in a malnourished patient who underwent emergency abdominal surgery.

## Case presentation

A 67-year-old man (weight, 51 kg; height, 162 cm) was referred to our hospital from a neighboring regional hospital because of an upper digestive tract perforation caused by a severe duodenal ulcer. He had pre-existing incomplete left hemiparesis due to an old cerebral infarction as well as a previous therapeutic history of diabetes mellitus. Blood chemistry results on admission revealed hyperglycemia (548 mg/dL) and a high Hb_A1c_ value (11.3%). He had not taken any medication for his diabetes for the entire previous year. His oral intake had been insufficient for several days. He appeared malnourished, and his serum albumin value was low at 2.6 g/dL.

He was scheduled to undergo emergency laparoscopic surgery for peritoneal leverage and direct closure of the perforated site. The proposed procedure was uneventfully completed in 129 min under general anesthesia. He was extubated after full recovery of consciousness, then transferred to the intensive care unit (ICU).

Although his respiration remained mostly stable with an oxygen face mask (5 L/min) while at rest, his coughing effort was limited owing to the insufficient relief of postoperative pain and exhausted respiratory muscle activities. On postoperative day (POD) 5, he experienced moderate dyspnea, and his SpO_2_ rapidly decreased to <90% despite an additional oxygen supply. A chest X-ray showed massive lung collapse of the left lower lobe. We treated him with frequent tracheal suction under direct visualization of his vocal cords using an Airway Scope™ (Pentax Co., Tokyo, Japan) as well as active postural drainage while he was sedated with dexmedetomidine. He subsequently resumed normal respiration with the disappearance of the atelectasis. We conducted respiratory support with noninvasive positive-pressure ventilation (NPPV) in continuous positive airway pressure (CPAP) mode at a PEEP of 8 cm H_2_O to prevent the recurrence of atelectasis. As PEEP was gradually decreased to 5 cm H_2_O, he sometimes complained of an uncomfortably fixed NPPV face mask. Therefore, he was weaned from NPPV on POD 7.

**Figure 1:**
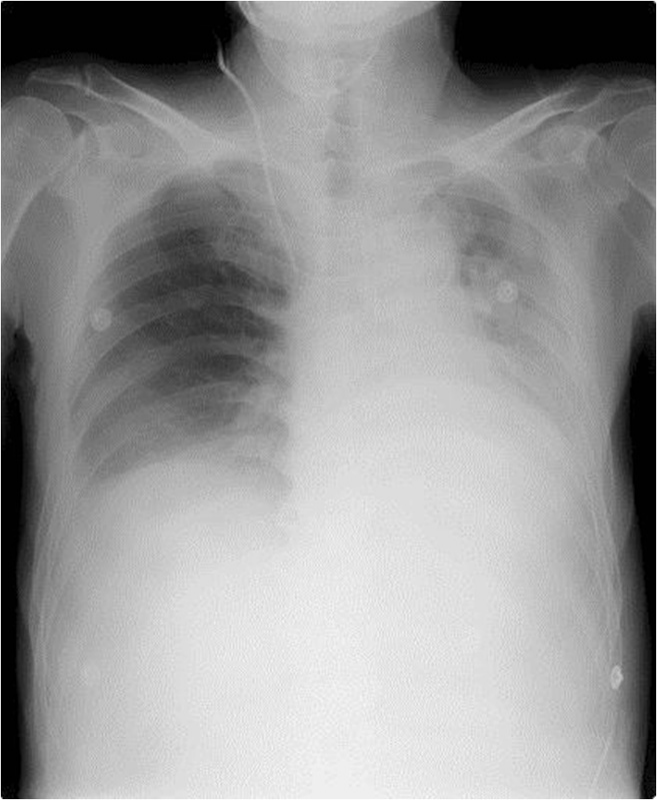
**Chest X-ray on POD 7 shows massive atelectasis in the left lower lobe.**

**Figure 2:**
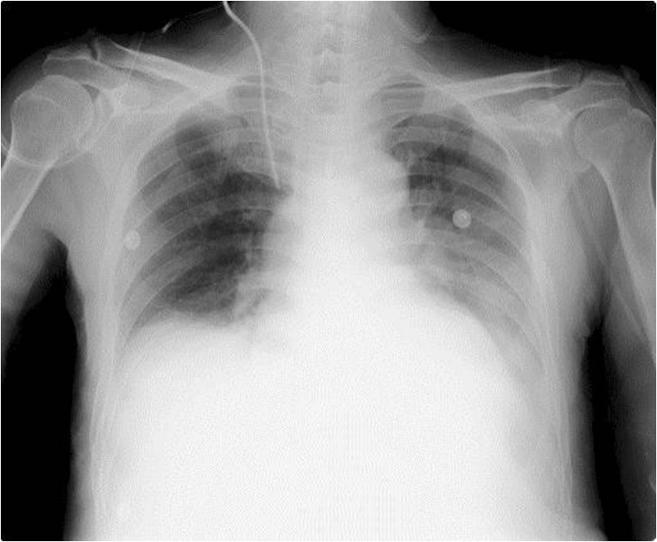
**Chest X-ray on POD 9 after NHF therapy was applied shows improvement of the atelectasis.**

Fifteen hours after the cessation of NPPV, his SpO_2_ unexpectedly decreased to <80%, and he complained of severe dyspnea. Chest X-ray showed the recurrence of massive atelectasis in the left lower lobe (Figure 1). Emergency bronchoscopic suction removed a mucous plaque that had extensively occluded the bronchial trees. We chose to alternatively apply NHF therapy at 40 L/min with an FiO_2_ of 0.40, delivering low-level positive airway pressure as respiratory assistance, rather than NPPV because we expected that the simple device might be more tolerable and easier to manage. The use of NHF therapy improved his respiration to a normal, regular pattern, and he comfortably breathed with inspiration of warmly humidified oxygen gases via his nose and expiration via his mouth. In addition to NHF therapy and active nutritional support, a physical therapist introduced bedside respiratory rehabilitation every day, which improved his coughing ability. Chest X-ray on POD 9 after the application of NHF therapy showed improvement of the atelectasis in the left lower lobe (Figure 2). NHF therapy was discontinued on POD 11, and the atelectasis did not recur throughout his remaining stay in the ICU until his discharge on POD 15.

## Conclusions

This patient experienced recurrence of massive postoperative atelectasis 15 h after NPPV support was terminated. We suspected that his nutritional condition was still in a serious catabolic phase from the preoperative period, even on POD 7. Therefore, his respiratory muscle activity had not recovered enough to remove the tracheobronchial secretions. In this patient, NPPV effectively prevented atelectasis by delivering 5 cm H_2_O of CPAP, which kept the peripheral airways open, as shown in a clinical study investigating the effect of NPPV on the prevention of postoperative atelectasis [1]. However, because he could not tolerate the face mask fixed to the NPPV circuit despite continuous sedation with dexmedetomidine, we chose to apply NHF therapy to assist respiration rather than perform a second trial of NPPV.

NHF therapy is a simple respiratory support technique that can supply heated, humidified oxygen via a large-bore nasal cannula at an extremely high flow rate of >30 L/min up to 60 L/min [2]. In pulmonary care practice, it is reportedly useful in the treatment of acute exacerbation among patients with chronic obstructive pulmonary disease because it washes out exhaled gases retained in the nasopharyngeal cavity by supplying gases with a low oxygen concentration at a high flow rate and promotes CO_2_ elimination [3]. Conversely, Parke et al. evaluated 15 surgical ICU patients and reported that NHF therapy at 35 L/min delivered 2.7 and 1.2 cm H_2_O of mean positive airway pressure with mouth closed and open positions, respectively [4]. Although these airway pressures were relatively low, NHF therapy maintained the delivery of positive pressure to the patient's airway in contrast to a conventional face mask. It is possible that even a low level of positive airway pressure of <5 cm H_2_O partially contributed to preservation of the patency of the peripheral airways, leading to the preventative effect against re-atelectasis.

NHF therapy has more advantages for postoperative patients requiring active physical therapy. Respiratory support with NHF therapy seems to be both less stressful and more tolerable for patients than NPPV, in which high positive pressure is continuously supplied through a fixed face mask. Therefore, patients do not require sedatives for the maintenance of NHF therapy unless they show mental disturbances before the introduction of NHF therapy. This patient tended to be more cooperative with the medical staff, including the physical therapist, under respiratory support with NHF therapy. When this patient adapted to the NHF therapy, he comfortably breathed with a normal regular pattern of inspiration via his nose and expiration via his mouth. Because optimally humidified gases are supplied at a high flow rate via the nose, NHF therapy may help to moisten mucous secretions and promote their clearance from the peripheral airways [5, 6]. The respiratory rehabilitation for this patient progressed smoothly and uneventfully along with the NHF therapy, and his coughing activity recovered enough to clear the tracheobronchial secretions.

In summary, we prevented the recurrence of massive atelectasis in a patient with malnutrition undergoing emergency surgery by conducting respiratory support with NHF therapy. NHF therapy at a flow rate of 40 L/min is thought to be an alternative respiratory support to NPPV set at less than 5 cm H_2_O of PEEP in terms of preserving the patency of the peripheral airways. In addition, postoperative respiratory rehabilitation progressed more smoothly under NHF therapy in this case, leading to the recovery of respiratory muscle activity. However, because the beneficial effects of NHF therapy on the respiratory system have not been fully confirmed in acutely ill patients, we should carefully monitor the sequential changes in the respiratory condition after the induction of NHF therapy.

## Consent

Written informed consent was obtained from the patient for publication of this case report and any accompanying images. A copy of the written consent is available for review by the Editor-in-Chief of this journal.
